# Lymphoepithelioma-Like Carcinoma of the Skin Treated with Wide Local Excision and Chemoradiation Therapy: A Case Report and Review of the Literature

**DOI:** 10.1155/2012/241816

**Published:** 2012-10-24

**Authors:** Theresa M. Gille, Edward F. Miles, Allen O. Mitchell

**Affiliations:** ^1^Department of Otolaryngology-Head and Neck Surgery, Naval Medical Center Portsmouth, 620 John Paul Jones Circle, Portsmouth, VA 23708, USA; ^2^Division of Radiation Oncology, Department of Radiology, Naval Medical Center Portsmouth, 620 John Paul Jones Circle, Portsmouth, VA 23708, USA

## Abstract

Lymphoepithelioma-like carcinoma of the skin (LELCS) is a rare cutaneous neoplasm microscopically similar to undifferentiated nasopharyngeal carcinoma. It is typically nonaggressive and is treated with wide local excision. However, we present a case of a patient with a regional recurrence and more aggressive LELCS with perineural invasion and positive margins for which he was treated with wide local excision followed by chemoradiation. We discuss the use of chemoradiation for this patient and review the literature, specifically pertaining to treatment of more aggressive cases of LELCS.

## 1. Introduction

Lymphoepithelioma-like carcinoma of the skin (LELCS) is an uncommon primary cutaneous neoplasm with less than 60 cases reported in the literature since it was first described by Swanson et al. in 1988 [1]. It has extremely low metastatic potential. Surgical excision is the preferred treatment although radiation has been used in cases which experienced a local recurrence or metastatic disease [1–3]. The use of radiotherapy is currently not well described for LELCS particularly in cases where there were positive margins or perineural invasion. We report a case of a gentleman with a regional recurrence and more aggressive LELCS showing perineural invasion and positive margins after excision. Due to the more aggressive nature of the tumor he was treated with chemoradiation postoperatively.

## 2. Case Report

**Figure 1:**
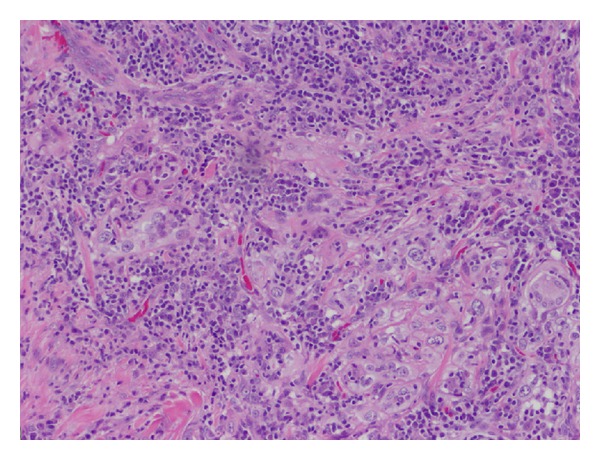
Hematoxylin and eosin stain (100x) showed aggregates of atypical epithelial cells and associated lymphoid tissue supported by fibrous connective tissue.

**Figure 2:**
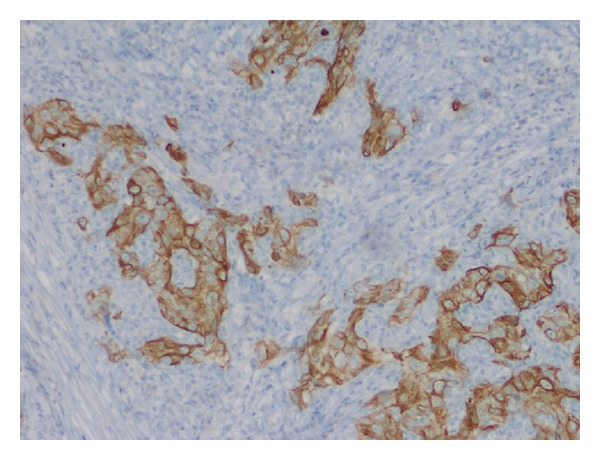
Immunohistochemical staining (100x) positive for CK-5/6.

A 76-year-old male presented to our clinic with a several-month history of a pea-sized lesion in his left upper lip which had been resected by another physician and found to be LELCS. The pathology report for the initial excisional biopsy resection did not comment on margin status. The case was presented to the institution's multidisciplinary tumor board and it was decided that close observation was warranted, rather than further treatment. Ten months later the patient returned with a similar lesion in his right cheek. Physical exam revealed a firm area superolateral to the nasolabial fold on the contralateral side of the face measuring 1.5 cm in largest dimension. It was slightly mobile with respect to the maxilla and overlying skin. He had no palpable lymphadenopathy and there was no palpable lesion in the left upper lip at the site of the original resection. A computed tomography (CT) scan showed a 2.8 × 2.6 cm soft tissue mass in the right premaxillary tissue and a positron emission tomography (PET)/CT scan showed increased metabolic activity in this tissue but not in the original site. In addition, the PET/CT also demonstrated a 2.2 cm × 2.4 cm ground glass opacity in the left lower lobe of the lung which also had increased metabolic activity. A CT-guided fine needle aspiration of the lung lesion was performed which showed malignant cells consistent with nonsmall cell lung carcinoma (insufficient tissue was recovered for further pathologic delineation). Staging studies showed this to be a T1a, N0, M0, Stage IA lung cancer. The patient underwent surgical excision of the new lesion in his right premaxillary space. The pathology revealed LELCS (Figure 1) with infraorbital nerve invasion and vascular invasion as well as positive superficial, medial and deep margins and an indeterminant lateral margin. Immunohistochemical staining was positive for pancytokeratin and CK-5/6 (Figure 2). Additional infra-orbital nerve was resected and was negative for tumor involvement. The patient was again discussed at our institution's tumor board and it was decided that, due to the recurrent nature of this disease, the more aggressive phenotype demonstrating perineural invasion, and the multiple positive margins, that he should receive adjuvant concurrent chemotherapy and radiation therapy to the tumor resection bed and margin as well as use intensity-modulated radiation therapy (IMRT) to track the infra-orbital nerve back to the skull base in the area of the trigeminal ganglion. He received 6,000 centigray (cGy) of a planned course of 6,600 cGy, with his course being truncated due to significant oral cavity mucositis. He received concurrent cisplatin (20 milligram per meter squared) weekly for five weeks. He was also referred to an outside institution for stereotactic body radiotherapy for the previously identified solitary lung lesion. He is disease-free two years following completion of his therapy.

## 3. Discussion

LELCS is a rare cutaneous neoplasm found most commonly in the sun-exposed skin of the head and neck although it has also been found on the trunk or extremities [1, 4] and is generally seen in elderly individuals [5]. Although the prognosis is generally good, there have been reports of metastases to lymph nodes at presentation [4] and local recurrence following surgical resection [1]. There is only one other case report in the literature which showed perineural invasion [6]. There has been only one reported death due to metastatic LELCS [1]. 

LELCS typically presents as a flesh-colored or red, firm nodule, plaque, or papule [5]. It is slow growing and generally asymptomatic and has usually been present in the patient from months to years [7]. Histologically, it is found in the mid to deep dermis and is nonecapsulated [7]. Histologically, LELCS is characterized by lobules, or nests of polygonal epithelioid cells surrounded by a dense infiltrate of T and B lymphocytes [5, 8] (Figure 1). These cytologic features are similar to those of metastatic nasopharyngeal carcinoma but are largely negative for Epstein-Barr virus (EBV) [5], although a recent case report noted an association of LELCS with EBV [9]. Immunohistochemical staining is positive for pancytokeratin and CK-5/6 (Figure 2) and p63 reactivity with the lymphoid infiltrate positive for CD3 and CD20 [5].

The differential diagnosis includes undifferentiated nasopharyngeal carcinoma, lymphoepithelial-like carcinoma metastasized from other sites, poorly differentiated squamous cell carcinoma, melanoma, lymphoma, and cutaneous lymphadenoma. It can generally be differentiated from undifferentiated nasopharyngeal carcinoma by the absence of EBV, notwithstanding the recent uncommon case report. It differs from squamous cell carcinoma in that it does not typically involve the overlying epidermis. Melanoma is positive for S100 and lymphoma cells will be positive for lymphoid markers. Cutaneous lymphadenoma is benign and does not have any cytological atypia.

Management should involve a complete head and neck exam to include evaluation of the nasopharynx to rule out metastasis from nasopharyngeal lymphoepithelioma [10]. Wide local excision has been the mainstay of treatment with radiation reserved for tumor recurrences [1, 2] or patients with lymph node metastasis at presentation [3]. There is only one other report of perineural invasion which was treated with Mohs micrographic surgery [6]. Mohs micrographic surgery has also been used as an alternative method for resecting LELCS based on the tendency of the tumor to recur following incomplete surgical excision [10, 11]. Due to the recurrent nature of the disease, the more aggressive phenotype demonstrating perineural invasion, and the multiple positive margins in this patient, aggressive adjuvant therapy with radiation and chemotherapy was felt to be indicated.
